# Valorization of Urban Street Tree Pruning Residues in Biorefineries by Steam Refining: Conversion Into Fibers, Emulsifiers, and Biogas

**DOI:** 10.3389/fchem.2021.779609

**Published:** 2021-11-15

**Authors:** Sebastian Hagel, Phillipp Lüssenhop, Steffen Walk, Satu Kirjoranta, Annalena Ritter, Carla Gabriela Bastidas Jurado, Kirsi S. Mikkonen, Maija Tenkanen, Ina Körner, Bodo Saake

**Affiliations:** ^1^ Institute of Wood Science, Chemical Wood Technology, Universität Hamburg, Hamburg, Germany; ^2^ Institute of Wastewater Management and Water Protection, Bioresource Management Group, Technische Universität Hamburg, Hamburg, Germany; ^3^ Department of Food and Nutrition, University of Helsinki, Helsinki, Finland; ^4^ Helsinki Institute of Sustainability Science, University of Helsinki, Helsinki, Finland

**Keywords:** tree pruning material, biorefinery, fibers, emulsions, biogas, steam refining

## Abstract

Street tree pruning residues are a widely available and currently undervalorized bioresource. Their utilization could help alleviate an increasing biomass shortage and offset costs of the pruning process for the municipalities. In this work, a holistic valorization pathway of pruning residues leading to fibers, oligosaccharides, biogas, and compost is presented. For this, representative mixtures of tree pruning materials from the most prevalent street tree genera (oak, linden, maple) found in Hamburg (Germany) were prepared by shredding and cleaning procedures. Collection of sample material was performed in summer and winter to account for seasonality. A steam-based fractionation was conducted using treatment severities ranging from log R_0_ = 2.5 to 4.0. At the highest severity, a fiber yield of around 66%, and liquor yield of 26–30% was determined. The fibers were evaluated with respect to their properties for paper product applications, with higher treatment severities leading to higher paper strengths. From the oligosaccharide-rich liquor, emulsions were created, which showed promising stability properties over 8 weeks of storage. The liquors and the rejects from the material preparation also displayed good potential for biomethane production. Overall, the differences between material collected in summer and winter were found to be small, indicating the possibility for a year-round utilization of pruning residues. For the presented utilization pathway, high severity treatments were the most promising, featuring a high liquor yield, good biomethane potential, and the highest paper strengths.

## Introduction

To reduce the dependency on fossil resources and the emission of carbon dioxide, a transformation of the current fossil-based economy into a sustainable bio-based economy is envisioned by governments worldwide (Dietz et al., 2018). Biorefineries, wherein the complete material is processed into new biomaterials or fuels will likely play a key role in the aforementioned transformation (Cherubini, 2010; Hassan et al., 2019). However, with increasing substitution of fossil resources, bioresource availability is expected to grow in importance (Lewandowski, 2015; Scarlat et al., 2015; Tzelepi et al., 2020). One way of mitigating a shortage of lignocellulosic material can be the mobilization of hitherto underutilized woody material sources, such as urban tree pruning materials accrued in maintenance measures. Such tree pruning activities are usually performed for safety and clearance reasons and are considered cost factors for the municipalities. A higher valorization of the pruning material could help offset these costs, which is mostly used for energy generation or merely discarded (McKeever and Skog, 2003; Oldenburger, 2010). As little is currently known about the valorization of tree pruning residues, the authors consider the examination of the quality and processing of this bioresource a worthwhile endeavor.

According to the tree register of Hamburg (Germany) (Behörde für Umwelt und Energie, 2020), a total of 223,201 trees can be found along the streets of the city. Of these, roughly 24% are linden (*Tilia* spp.), 22% are oak (*Quercus* spp.), and 14% are maple trees (*Acer* spp.). About 100 other genera make up the remaining 40% of the street trees, with no singular genus amounting to more than 5%. These numbers are in accordance with studies showing that often a few genera account for the majority of the street trees, with maple, oak, and linden trees being among the most numerous genera found in urban areas (Pauleit et al., 2002; Sjöman et al., 2012; Yang et al., 2015). Tree pruning residue differs significantly from conventionally used stem wood, as it consists, to a large extent, of twigs and branches (Thrän et al., 2016). Due to dimensional differences, a higher content of bark is to be expected in branch wood. Bark consists of a lower amount of carbohydrates and a higher amount of lignin, extractives, and mineral compounds than stem wood (Hakkila, 1989). Furthermore, branches are rich in tension wood, which differs significantly from nontension wood (Ruelle, 2014).

Steam-based treatments, such as steam explosion or steam refining, lend themselves to the processing of locally incurring, moist, and low-value biomass, such as pruning residues, as energy-intensive dewatering procedures are not needed. Additionally, no chemical recovery is needed, and corrosion is minimized, which leads to low initial investments and operational costs (Garrote et al., 1999) and a high environmental friendliness (Islam et al., 2020). In steam-based treatments, the biomass is treated with high-pressure steam at elevated temperatures. The water dissociates and creates an acidic milieu in the microporous structure. Subsequently, acetyl groups are cleaved from the hemicelluloses, and the emerging acetic acid increases the hydronium ion concentration further catalyzing the hydrolysis reactions of the polysaccharides (Garrote et al., 1999). As the polysaccharides are depolymerized, they start to solubilize into the water in the form of mono- and oligomeric pentoses and hexoses (Bobleter, 1994). Upon further steam treatment, hexose molecules are dehydrated to 5-(hydroxymethyl)-2-furaldehyde (5-HMF) and then further degraded into formic acid and levulinic acid, while pentoses are degraded to furan-2-carbaldehyde (furfural), following various reaction routes (Rasmussen et al., 2014). To follow changes in the main components of the lignocellulosic biomass during steam treatments, the use of the so-called severity factor (log R_0_), which combines the two main reaction parameters, treatment duration (t) and temperature (T) according to Eq. 1, has been established (Chornet and Overend, 1991).

$$\log R_{0}=\log\left(t\;\times \;e^{\frac{\left(T-100\right)}{14.75}}\right)$$ (1)

Following the steaming treatment, a mechanical separation of the material is carried out, either by a sudden pressure release, as is the case in the steam explosion process (Focher et al., 1991) or by refining of the material (Schütt et al., 2012). The resulting material is a mixture of solid, fibrous material, and solubilized oligo- and monosaccharides, as well as of different degradation products. The fractionation of different lignocellulosics, such as poplar (Schütt et al., 2011), spruce forest residues (Janzon et al., 2014), corn stover (Krafft et al., 2020a), maize silage (Krafft et al., 2020b), or waste medium density fiberboards (Hagel and Saake, 2020) by steam refining, have been investigated before. Often, steam treatments are used to increase the accessibility of the lignocellulosic structure for enzymatic hydrolysis with subsequent fermentation for bioethanol production. In such cases, high treatment severities of four or higher are optimal (Limayem and Ricke, 2012; Zabed et al., 2016). Alternatively, the fibers might be useable in paper applications after further processing (Kokta and Aziz, 1998; Hagel et al., 2021). As the demand for paper-based packaging material is expected to remain high due to the surge in e-commerce, and hardwood fibers are known to be short and stiff in comparison with softwood fibers (Twede et al., 2015), an incorporation of the fibers in the production of corrugated paperboard could be an environmentally friendly and economically viable utilization of the fibers. The oligosaccharide-rich liquor, on the other hand, could be of great interest for major industries (e.g., cosmetic, pharmaceutical, food) as a sustainable produced stabilizer for emulsion systems, which are trying to substitute nonrenewable chemicals (Mikkonen, 2020). Additionally, the use of biogas plants to process side stream material and residues has been suggested before (Dieckmann et al., 2018) and offers a well-established and robust method of processing otherwise hard to valorize material streams. Other reported possible uses for steaming fractions include nanofibrillated cellulose (Moon et al., 2011), prebiotics (Singh et al., 2015), or films and coatings (Mikkonen and Tenkanen, 2012; Egüés et al., 2013), though those are not further considered in this study.

**FIGURE 1:**
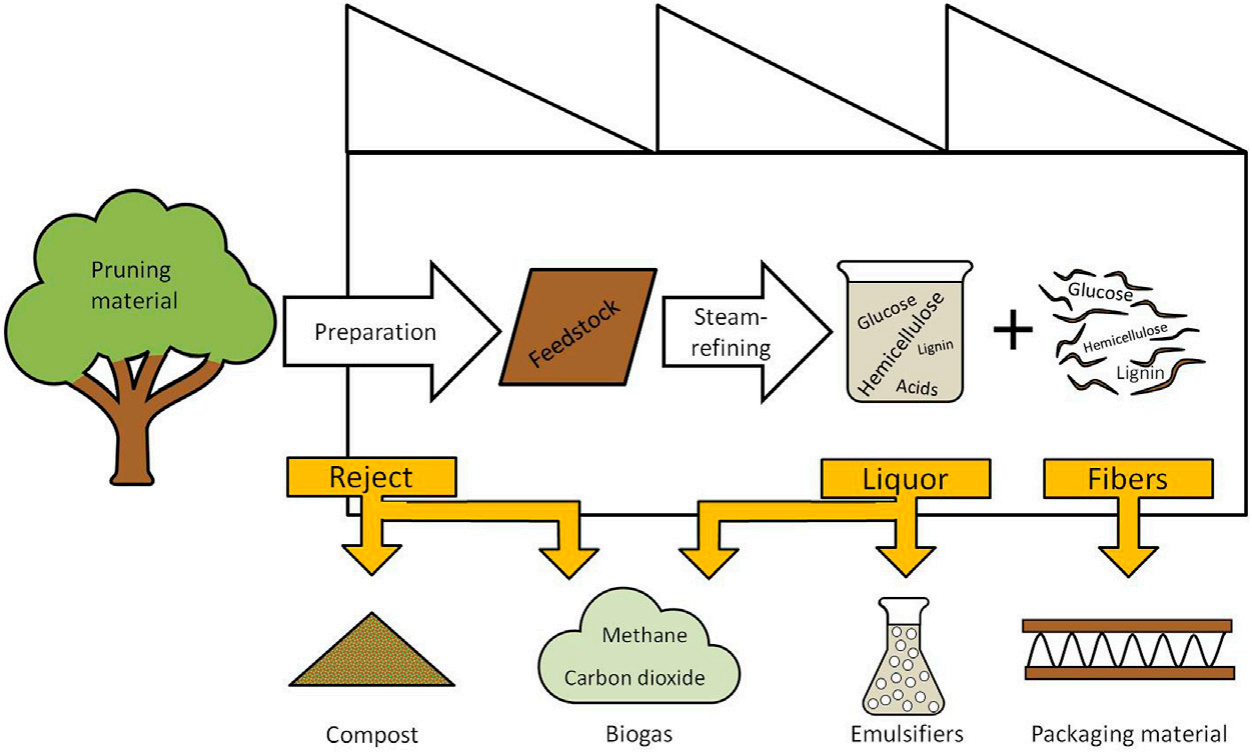
Flowchart of the tree pruning residue processing zero-waste biorefinery.

The aim of the present study is to evaluate a complete utilization of tree pruning residue within the context of a biorefinery (Figure 1). For this purpose, material of the three main genera (*Tilia* spp., *Quercus* spp., and *Acer* spp.), found along the streets of Hamburg (Germany), was collected to generate a representative mixture. The raw material was collected in winter and summer to account for changes in the pruning material induced by seasonal changes (i.e., foliage, biological activity). The street pruning material was sieved and washed to prepare for the steam-refining treatment. The steam treatments were performed on both sample sets using severities ranging from 2.5 up to 4.0; as for steam treatments of hardwoods with a severity higher than log R_0_ = 4, an increasing degradation of the dissolved oligosaccharides and a subsequent loss of yield has been reported (Li et al., 2005; Castro et al., 2013). The resulting fibers and liquors were characterized concerning carbohydrate composition, lignin content, and main degradation products. From the fibrous fraction paper, test sheets were generated, and the tensile-, tear-, and compression strength as well as the brightness were measured to assess a possible application of the fibers in paper products. The liquor was purified, and the emulsion-forming ability and long-term stability were evaluated. The biogas potential was measured from the potential reject fractions of the process cascade and from the liquor. Using the data generated, process balances were calculated, and a flowchart of an exemplary zero-waste valorization pathway was created with fibers, emulsifiers, biogas, and compost as products.

## Material and Methods

### Raw Material Collection and Preparation

Of the three main tree genera (*Tilia* spp., *Quercus* spp., and *Acer* spp.) grown along the streets of Hamburg (Germany), the sample material was cut from the tree crowns. These branches and twigs were chipped on-site using standard machinery of the tree maintenance companies. Samples collected in March were named winter samples and featured no leaves, while samples collected between June and August were named summer samples and included leaves.

In order to get a defined particle size, the pruning residues were sieved for 5 min in a vibrating sieve at 220 rpm. The fine fraction passed a 4 mm × 4 mm grid sieve, and the coarse fraction was retained by a 10 mm slit sieve. Both were disregarded as rejects. To remove further sand and dust particles still sticking to the accept fraction, the material was washed using a cylindrical washing drum (h = 38 cm, *Ø* = 40 cm) with a grid size of 4 mm × 4 mm. The drum was rotated for 10 min at 30 rpm. After 3 min sedimentation, the washed material was removed from the washing water.

For the steam refining treatment, a representative mixture from the three samples was produced using 40% of linden, 37% of oak, and 23% of maple material (based on dry matter), corresponding to the distribution of the three most common genera found in the tree register of Hamburg (Behörde für Umwelt und Energie, 2020).

### Raw Material Characterization

From each of the original tree samples, three random batches were drawn and sorted by hand into macrocomponent categories: wood chips, leaves, bark, branches, seeds, and miscellaneous for material that did not fit into the previous categories or could not be identified. Dry matter (DM) and water content of original samples and fractions were measured as described by Walk et al. (2021), Volatile solids (VS) and ash content were measured according to TAPPI T211 om-16. Basic elements (carbon, nitrogen, hydrogen, sulfur) were determined using an NCHS-Analyzer Vario Macro Cube (Elementar, Langenselbold, Germany). Oxygen was calculated as mass balance considering the basic elements and the ash. For the determination of the extractives, washed material was milled (SM 2000; Retsch, Haan, Germany) to a particle size of ≤1 mm diameter and extracted with an ASE 350 (ThermoFisher Scientific, Waltham, MA, United States). An extraction sequence using petroleum ether (70°C), acetone/water (9:1, 70°C), and water (90°C) with a pressure of 10 MPa was conducted. For the *Tilia* spp. samples, due to the generation of a gelatinous mass, which clogged the filter, the water extraction step was replaced with an extraction in a Soxhlet apparatus. The material was cooked in distilled water for 45 min and subsequently extracted for 3 h. Carbohydrate and lignin content determination was performed as described in the *Chemical characterization of the fibrous and liquor fractions* section.

### Steam-Refining Treatment

To follow the changes in the main components of the lignocellulosic biomass during steam treatments, the use of the so-called severity factor (log R_0_), which combines the two main reaction parameters, treatment duration (t) and temperature (T) according to Eq. 1, has been established by Chornet and Overend (1991).

The steam-refining treatments were conducted on batches of 300 g (of calculated DM) of the washed material mixtures in a 10-L reactor by Martin Busch and Sohn GmbH (Schermbeck, Germany), using six different severity grades (log R_0_/°C/min): 2.5/150/10, 2.8/150/20, 3.1/160/20, 3.4/170/20, 3.7/180/20, and 4.0/190/20. In the final 30 s of the treatment, a mechanical defibration was carried out by rotation of a four-bladed system along the bars mounted to the interior of the reactor. The fiber slurry was removed from the reactor and dewatered by centrifugation in a spin dryer, separating the liquor from the fiber fraction. A more thorough description of the procedure is given by Hagel and Saake (2020). Subsequently, the fiber material was passed three times through a 12” Sprout-Bauer laboratory refiner (Andritz, Graz, Austria). The first pass was done at a gap distance of 0.5 mm at a DM content of 4%. For the following two passes, the gap distance was reduced to 0.2 mm.

### Chemical characterization of the fibrous and liquor fractions

For monomeric carbohydrate and lignin content determination, acid hydrolysis was conducted. The raw material after extraction and the fibrous samples were milled and treated by a two-stage acid hydrolysis using sulfuric acid (72%), while the freeze-dried liquor samples and the soluble and insoluble fractions after centrifugation (see the *Emulsion Preparation and Stability Testing* section) were treated by a one-stage acid hydrolysis as described by Lorenz et al. (2016). The hydrolyzation in the autoclave was conducted at 120°C and 0.12 MPa for 30 min. The hydrolyzed samples were filtered on a G4-sintered glass crucible using distilled water. The acid-insoluble residue (comparable with Klason lignin) caught in the filter was dried at 105°C, and the weight was gravimetrically measured. To determine the monomeric carbohydrate content, borate-anion exchange chromatography was performed on the hydrolyzed filtrates using a Dionex Ultimate 3000 (ThermoFisher Scientific, Waltham, MA, United States) with anion exchange resin MCI GEL CA08F (Mitsubishi Chemical, Tokyo, Japan) in a 5 mm × 120 mm column packed at 65°C as described by Lorenz et al. (2016). Additionally, the acid-soluble lignin content was measured from the hydrolyzed filtrates with a UV-Spectrophotometer LAMBDA 650 (PerkinElmer, Waltham, MA, United States) at 205 nm wavelength as described by Maekawa et al. (1989) for the raw materials.

Furfural and 5-HMF determination from the liquor was done by reverse-phase high-performance liquid chromatography immediately after separation, using an Aquasil C18 column (250 mm × 4.6 mm) (ThermoFisher Scientific, Waltham, MA, United States) conditioned to 25°C. Acetonitrile (Mallinckrodt Baker Bv, Deveneter, Netherlands) and 1 mM phosphoric acid (Riedel-da Haen, Seelze, Germany) in different concentrations were used as eluents with a flow rate of 1 ml/min as described by Krafft et al. (2020a). UV detection was done at 280-nm wavelength.

### Pulp Characterization

Additional beating was conducted in a Jokro mill (FRANK-PTI, Birkenau, Germany) on all fiber pulps. The beating degree was determined with a Schopper–Riegler freeness tester type SR1 (Karl Schröder KG, Weinheim, Germany). Paper sheets of about 75 g/m^2^ were produced from the pulp with a Rapid-Köthen sheet-forming machine (FRANK-PTI, Birkenau, Germany). Conditioning was done for 24 h at 23 ± 1°C and 55 ± 2% relative humidity before physical testing. Short span compressive (SCT) strength, tensile strength, and tear strength were measured according to DIN 54518:2004, ISO 1924-2:2009, and ISO 1974:2012-09, respectively, using instruments manufactured by FRANK-PTI Gmbh (Birkenau, Germany). Index calculation was done according to TAPPI T220. The brightness was measured according to TAPPI T525 with an ELREPHO 450X from Datacolor (Rotkreuz, Switzerland).

### Emulsion Preparation and Stability Testing

Freeze-dried liquors from winter and summer pruning samples steam treated at a severity of 3.7 were purified from undissolved particles using two different methods: centrifugation and filtration, after the liquor powders were mixed in deionized water at 5% (w/w) by magnetic stirring for 2 h. The suspensions were either filtered using a glass microfiber filter having a pore size of 1.6 µm (GF/A, Whatman^®^, Merck, Darmstadt, Germany) or centrifuged with 9,000 × *g* for 20 min (Z 323, Hermle Labortechnik, Wehingen, Germany). The DM content of the soluble fraction, the supernatant, was calculated based on the DM content of the precipitant or material on the filter, dried in a vacuum oven at 50°C for 24 h before weighing. Samples from the supernatants were collected and freeze dried. Carbohydrate and acid hydrolysis residue determination was conducted on the soluble and insoluble fraction as described in the *Chemical Characterization of the Fibrous and Liquor Fractions* section.

Emulsions were prepared using the centrifuged supernatants or filtered solutions after diluting them to the desired concentration (1 w% in the final emulsion). Hexadecane (1 w% in the final emulsion) was weighed and added on top of the solutions. Coarse emulsification was conducted by mechanical mixing using an Ultra Turrax (T-25 Basic, IKA, Germany) bench-top homogenizer at 11,000 rpm for 2 min. The emulsions were further homogenized by circulating them for 2 min with a Microfluidizer 110Y high-pressure homogenizer (Microfluidics, Westwood, MA, United States) at 800 bar. Approximately 150 ml of emulsion was collected from each experiment. After emulsification, sodium azide 0.02% (w/w) was added to avoid microbial growth.

Droplet size distribution analysis was conducted with fresh emulsions and after 1, 2, 5, and 8 weeks of storage at room temperature, using static light scattering (Mastersizer 3000, Malvern Panalytical, United Kingdom), with the refractive index value for hexadecane (1.434). The emulsions were gently mixed before the analysis by turning them upside down for 10 times. Emulsion stability was also characterized by Turbiscan^™^ LAB Stability Analyzer (Formulaction SA), measuring the transmission and backscattering of light in samples stored in glass vials, avoiding mixing. A thorough explanation of the working principle of Turbiscan is given by Bhattarai et al. (2020).

### Biochemical Methane Potential and Biodegradability

Biogas tests on three sets of substrates were conducted: 1) leaves, 2) fine sieving rejects, and 3) liquors of the summer and winter samples treated at severities of 2.5, 3.4, and 4.0. The substrates consisted of material mixtures of the three tree species as indicated in the *Raw Material Collection and Preparation* section. Additionally, the leaf samples were investigated separately for each tree genera.

For the leaves and reject, a eudiometer test was conducted. For the liquors, an AMPTS^®^II test system (BPC Instruments, Lund, Sweden) was used. For all tests, the methodology presented by Holliger et al. (2021) was followed. The substrate-to-inoculum ratio was 0.5 based on volatile solids (VS), and the temperature was set at 37°C. The pH was measured initially to ensure the range between 6.8 and 7.2. The termination criteria for the test was chosen according to Holliger et al. (2021), which requires that the volumetric methane production of the last 3 days of experiment is below 1% of the total accumulated volume produced.

The measurement of methane (CH_4_) is automated in the AMPTS system by an integrated carbon dioxide (CO_2_) fixation reagent (NaOH). For the eudiometer test, biogas composition was measured frequently and normalized to obtain the shares of CH_4_ and CO_2_. For both test systems, biochemical methane potential (BMP) was calculated based on both VS and fresh matter (FM) initially added. Biodegradability (BD) was calculated in two ways; 1) by dividing the experimental (exp.) BMP by the theoretical (th.) BMP, 2) by dividing normalized CH_4_ and CO_2_ masses by initial VS added. Theoretical BMP was calculated according to the Buswell–Boyles equation (Boyle, 1977). A detailed description of the procedure and calculations can be found in Walk et al. (2021).

## Results and Discussion

### Raw Material Characterization

To quantify differences in the overall composition of typical tree pruning material in comparison with conventionally used wood chips, the material was inspected and sorted by hand into six different fractions (leaves, wood, bark, branches, seeds, and miscellaneous). The macro composition of the tree pruning material collected from the three tree genera in summer is given in Table 1. While the differentiation of the residue fragments is not always distinct, especially in the case of bark and branches, it can be said that the wooden components (wood and branches) make up the majority of the material in all collected samples. Overall, the amount of bark (separated and clinging to the branch wood) in tree pruning residue is high in comparison with conventional used wood chips from stem wood due to the dimensional differences of the material. Also, as the nonwooden components (bark, leaves, seeds, and miscellaneous small fragments) account for a non-negligible part of the material, the amount of wooden material in the tree pruning residue is lower than in conventional wood chips. Naturally, due to seasonal growth periods, a lower amount of leaves and seeds was found in the winter pruning material.

**TABLE 1:** Composition of the tree pruning material collected in the summer [(% w/w), mean (SD), *n* = 3].

	Leaves	Wood	Bark	Branches	Seeds	Misc
Oak	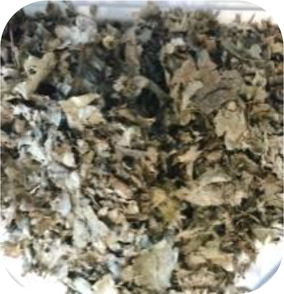	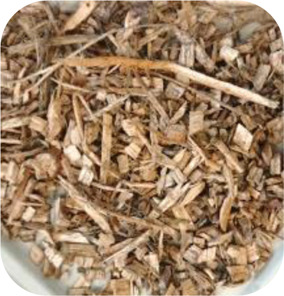	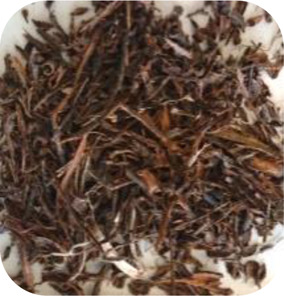	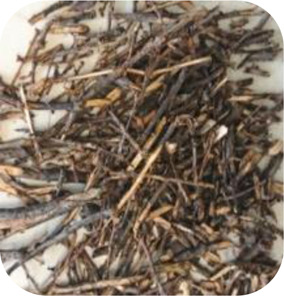	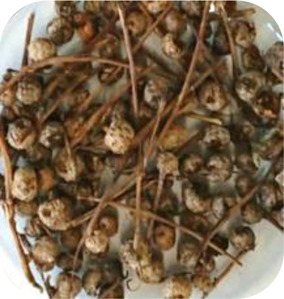	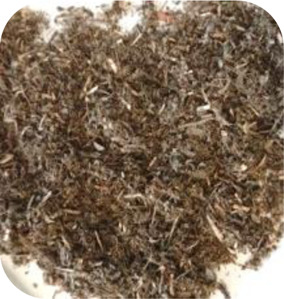
	17.6 ± 0.3	39.5 ± 3.0	11.1 ± 1.4	6.8 ± 1.6	0.8 ± 0.2	24.1 ± 4.6
Linden	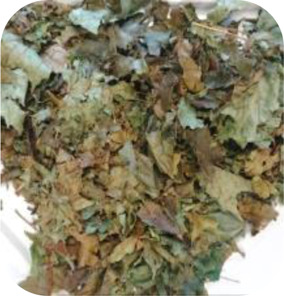	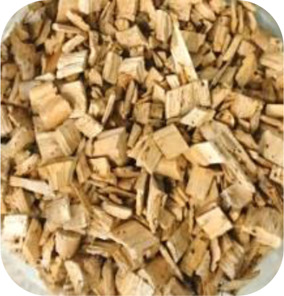	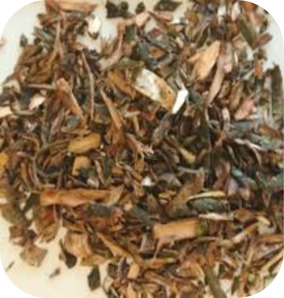	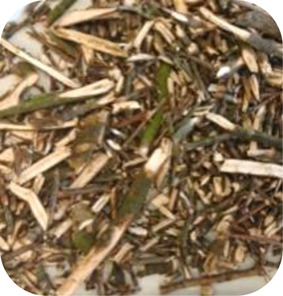	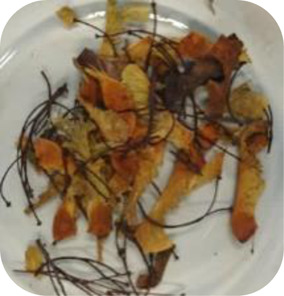	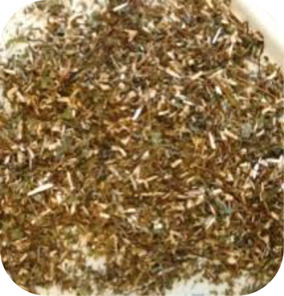
	19.9 ± 2.2	28.9 ± 1.1	27.0 ± 0.4	5.5 ± 0.9	1.2 ± 0.1	17.5 ± 2.7
Maple	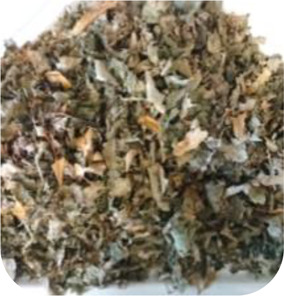	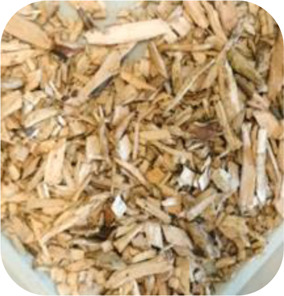	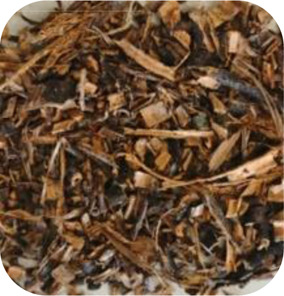	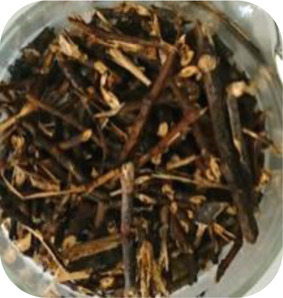	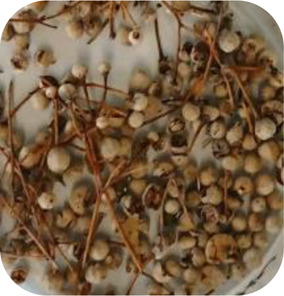	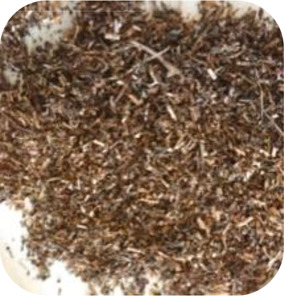
	7.4 ± 0.9	32.8 ± 3.7	7.5 ± 0.3	39.2 ± 2.8	0.5 ± 0.1	12.7 ± 1.6

In order to further characterize the material, the content of the extractives, ash, carbohydrates, and lignin was determined on the accepted material of the three tree genera samples collected in summer and winter separately (Table 2), as the asserted anatomical differences between conventional wood and tree pruning wood also influence the chemical composition of the material, which can be important to know how to facilitate an optimal utilization. In comparison with pure wood samples of oak (Ghavidel et al., 2020), linden (Kusiak et al., 2020), and maple (Výbohová et al., 2018), the content of carbohydrates found in the pruning residue is lower, while the amount of extractives and ash is higher. The main reason for this circumstance is most likely the high content of bark found in the samples, which features a low amount of carbohydrates and a high amount of extractives and ash (Hakkila, 1989). Furthermore, it has been reported that high environmental stress, such as found in urban street environments, can lead to an increase in extractive components (Gershenzon, 1984) and a reduction in cellulose and lignin in the xylem (Kusiak et al., 2020). No meaningful differences in chemical composition between samples collected in summer and winter could be discerned.

**TABLE 2:** Content of extractives, ash, carbohydrates, and acid hydrolysis residue of tree pruning material harvested in winter and summer (% based on raw material).

		Winter harvested				Summer harvested			
		Oak	Linden	Maple	Mixture	Oak	Linden	Maple	Mixture
Extractives	P-ether	0.7	5.2	0.4	2.5	0.8	4.3	1.0	2.3
	Acetone/H_2_O	3.0	6.6	2.5	4.3	2.6	2.8	6.3	3.5
	H_2_O	4.0	2.4	3.8	3.3	2.8	1.1	3.0	2.2
	∑	7.7	14.2	6.7	10.1	6.2	8.3	10.3	8.0
Ash	∑	4.9	4.4	4.2	4.5	3.5	4.8	4.1	4.2
Carbohydrates	Glucose	31.2	29.0	32.2	30.5	34.7	31.3	27.5	31.7
	Xylose	14.3	11.1	14.4	13.0	12.8	10.8	12.8	12.0
	Mannose	0.7	1.2	1.1	0.9	0.9	1.3	1.0	1.1
	Galactose	1.4	1.8	1.2	1.5	1.8	1.4	1.2	1.5
	Arabinose	1.3	2.5	1.4	1.8	1.4	2.1	1.7	1.7
	Rhamnose	0.4	0.8	0.5	0.6	0.5	0.7	0.6	0.6
	∑	49.4	46.1	50.7	48.4	52.2	47.6	44.8	48.6
Residue	Acid soluble	2.4	2.3	2.6	2.4	2.3	2.0	1.9	2.1
	Insoluble	25.9	23.0	26.3	24.8	23.7	21.3	24.1	22.8
	∑	28.4	25.2	28.9	27.2	26.0	23.3	26.0	24.9

### Composition of the Material Fractions After Steam Refining

**FIGURE 2:**
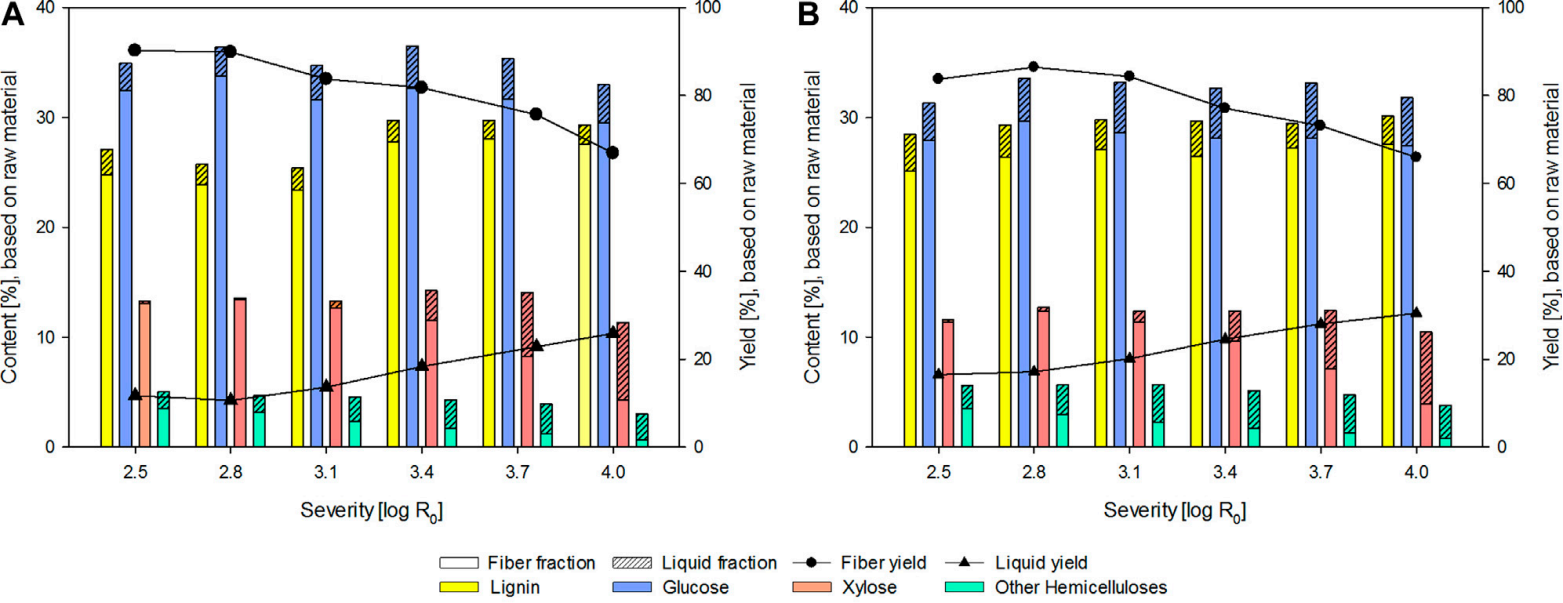
Yield and composition of the fibers and liquor after steam refining of sample material collected in winter **(A)** and summer **(B)** (% based on raw material).

The yield and composition of the fibers and the liquors after steam refining were determined and plotted against the severity of the treatment (Figure 2). Following an increase in treatment severity, a decrease in fiber yield and increase in liquor yield can be observed for both the winter and summer sample sets. In comparison with industrial poplar wood chip steam refined using identical treatment severities (Hagel and Saake, 2020), the yields determined for the fiber fraction are lower, and the yields of the liquors are higher for the tree pruning residues. This is likely due to the low amount of fiber containing material and the high amount of bark, as the bark contains a large amount of readily extractable components (Hakkila, 1989).

At the lowest treatment severity, a surprisingly high amount of solubilized glucose can be determined. The found glucose likely stems from the phloem of the branches, as the cellulose itself is highly resistant to hydrolytic cleavage due to its high crystallinity (Puls et al., 1985). At higher treatment severities, the cell wall components, in particular the hemicellulose, increasingly hydrolyze and subsequently solubilize. This behavior is reflected in the decrease in hemicellulose content in the fiber fraction and the concurrent increase in the liquor. The pH of the liquor decreases to around 4, 5 at the highest severity as acetic acid is increasingly cleaved from the hemicelluloses. At the higher treatment severities, the solubilized hexoses and pentoses are increasingly degraded into furfural and 5-HMF. This can be observed for the winter and summer samples in similar amounts (Supplementary Data File). In the severity range studied, the solubilization of polysaccharides proceeds faster than the concurrent degradation reactions. Thus, the amount of hemicelluloses in the liquid phase rises continuously. A slight increase in total lignin of the fiber and liquor in comparison with the raw material can be observed, which can be explained with the generation of pseudo-lignin, a term used for agglomerated carbohydrate and lignin degradation products, which are acid insoluble (Hu et al., 2012).

Besides the hydrolysis-related chemical changes in the material, the shearing forces applied in the refining process defibrate the material and cause morphological changes. After steam-refining treatments with a low severity, a high amount of fiber bundles is visible, and by increasing the treatment severity, the fibers are more evenly separated as the material softens (Supplementary Figure 1). Overall, no distinct difference between the morphology of fibers produced from the material collected in summer and winter can be observed visually.

### Paper Test Sheet Properties

**FIGURE 3:**
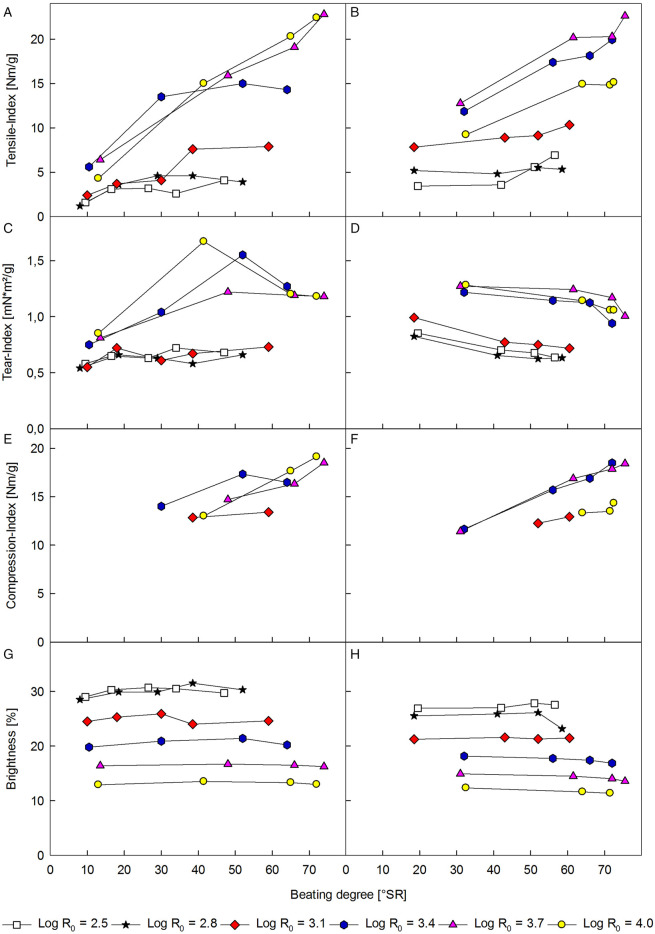
Comparison of the tensile **(A,B)**, tear **(C,D),** and compression indices **(E,F)** and brightness **(G,H)** of paper test sheets made from steam-treated tree pruning fibers harvested in winter **(left row)** and summer **(right row)**.

The fibers generated by the steam refining were further processed by additional refining and subsequently beaten to beating degrees ranging from around 10 to 75 SR. In Figure 3, the tensile, tear, and compression indices, as well as brightness of paper test sheets produced from the summer and winter fiber sample steam refined at severities ranging from Log R_0_ = 2.5 to 4.0 and plotted over the beating degree is depicted. A distinct influence of the severity of the steam treatment on all evaluated parameters can be observed for the winter and summer samples. Looking at the tensile (Figures 3A, B), tear (Figures 3C, D), and compression indices (Figures 3E, F), an increase in treatment severity also led to an increase in strength properties for treatment severities higher than Log R_0_ = 3.4, while almost no influence on the properties at lower severities was found. The compression strength (Figures 3E, F) from test sheets produced from fibers treated at low severities of Log R_0_ = 2.5 and 2.8 were not measurable due to their low values. The steam treatment at high severity can lead to a softening of the fibers (Kokta, 1991), which might enhance the defibration as well as the internal and external fibrillation in the refining step. While for the winter sample the highest tensile, tear, and compression strengths were achieved with the fibers treated at the most severe conditions, the indices of the summer samples started to decrease again for the highest treatment severity of Log R_0_ = 4.0. This could be due to negative effects of the increasing hydrolysis of the hemicelluloses and a beginning depolymerization of the cellulose on the paper properties. An increase in treatment severity also led to slightly darker fibers caused by the formation of chromophoric groups (Macdonald and Franklin, 1969), as indicated by the decreasing brightness (Figures 3G, H).

Compared with test sheets made from recycling corrugated paperboard (Hagel et al., 2021), the tear and tensile indices produced from the steamed pruning residues are low, while the compression indices are comparable with the values determined for recycled liner and recycled medium in cross-direction (Adamopoulos et al., 2007). As the compression index is a good indicator for the edgewise compression strength (Dimitrov and Heydenrych, 2009), which is an important parameter for packaging paper (Whitsitt, 1985; Markström, 1999), a utilization of the steam-treated pruning residues fibers as reinforcement or filler material in the fluting of recycled corrugated paperboard might still be a viable valorization pathway, especially as the dark color of the fibers is not necessarily detrimental in such applications, and an additional bleaching is unlikely to be economically sensible. While the differences in paper properties from steam-refined fibers from tree pruning residues collected in summer and winter are slight, the optimal conditions do vary. Going forward, further research into the reasons for these differences and optimal preparation and steam treatment conditions should be conducted.

### Utilization of Polysaccharides as Emulsifier

**FIGURE 4:**
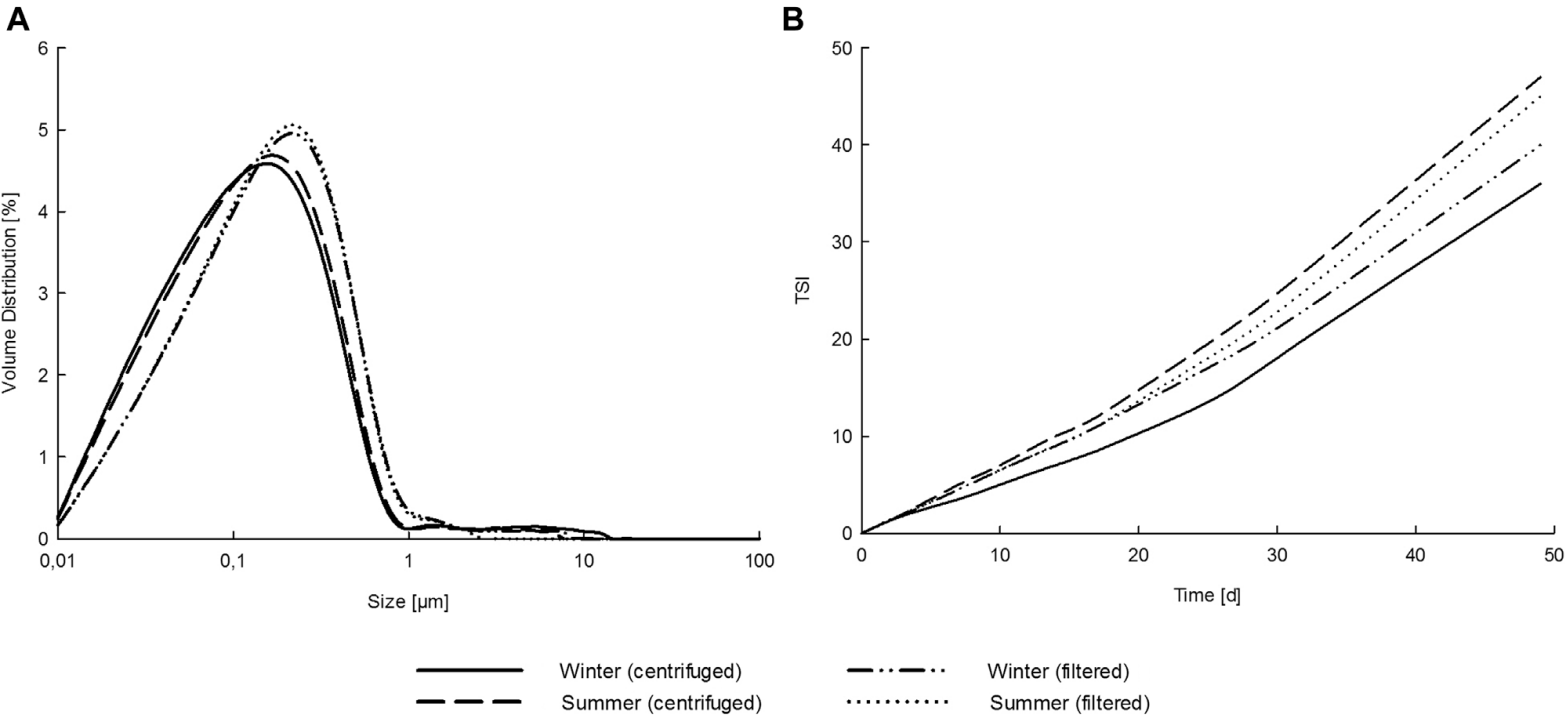
Droplet size distribution after 8 weeks of storage **(A)** and Turbiscan stability index (TSI) as a function of storage time **(B)** of tree pruning residue liquor–hexadecane emulsions.

Both filtration and centrifugation feasibly removed insoluble particles from the liquor suspensions. During centrifugation, 11.0 and 13.1% solids were precipitated from winter and summer samples treated at a severity of 3.7, respectively. Filtering removed 11.4 and 14.5% of the corresponding solids (Supplementary Data File). Purified and diluted liquors formed stable emulsions with unimodal droplet size distribution (Figure 4A) and volume average droplet size D (4.3) in a range of a 100 nm (Table 3). Liquors from winter and summer pruning samples yielded similar emulsions. Foaming of the liquor during emulsification was visually observed with all samples.

**TABLE 3:** Droplet size (µm) of the emulsions during storage.

Sample	Time (weeks)	Dx (50)	Dx (90)	D [4.3]	D [3.2]
Winter filtered	0	0.07	0.20	0.09	0.049
	1	0.09	0.33	0.14	0.059
	2	0.11	0.38	0.17	0.068
	5	0.13	0.44	0.22	0.074
	8	0.16	0.51	0.27	0.088
Winter centrifuged	0	0.06	0.16	0.08	0.045
	1	0.09	0.31	0.22	0.056
	2	0.09	0.31	0.25	0.059
	5	0.11	0.38	0.28	0.065
	8	0.12	0.42	0.28	0.070
Summer filtered	0	0.07	0.21	0.09	0.048
	1	0.09	0.31	0.14	0.058
	2	0.10	0.35	0.15	0.063
	5	0.14	0.44	0.20	0.079
	8	0.16	0.49	0.23	0.088
Summer centrifuged	0	0.05	0.13	0.07	0.043
	1	0.08	0.27	0.16	0.055
	2	0.09	0.30	0.18	0.058
	5	0.11	0.37	0.22	0.067
	8	0.13	0.42	0.23	0.073

Note. Dx (50) and Dx (90) values indicates that 50% and 90%, respectively, of the droplets are below the size. Average droplet size was calculated based on volume [D (4.3)] and surface area [D (3.2)].

The Turbiscan stability index of all emulsions steadily increased during storage reaching values between 30 and 50 (Figure 4B), which indicates some instability. Together with visual observations, increased backscattering on the top of the sample vials and increased transmission on the bottom indicated that creaming was the main destabilization mechanism. The droplet size, determined by static light scattering after a gentle mixing of the emulsions each time, was stable during the 8 weeks of storage. This indicates that notable coalescence or flocculation did not occur. Creaming may be considered a low risk to emulsion stability compared with other destabilization mechanisms, as the droplet morphology was maintained. The effect of the presence of insoluble fractions in biomass extracts on emulsion stability is not straightforward, and should be further considered and optimized (Bhattarai, 2020). A future strategy could involve using more concentrated emulsions or other ways to increase emulsion viscosity, to overcome creaming. The emulsification and storage stability evaluation illustrated a promising process to valorize the oligosaccharide-rich liquors from tree pruning fractions and yield functional ingredients, e.g., for chemical industry.

### Biomethane Potential and Biodegradability

Table 4 shows the results of the biogas tests conducted with the fine sieving reject, leaves, and liquors. The experimental BMP_VS_ was 25%–58% lower than the theoretical BMP_th_, since the latter is a simplification based on elemental composition and not differentiating between chemical compounds (*Biochemical Methane Potential and Bbiodegradability*). The BD_th_ is based on the latter. It was higher for the liquors (56–74% of BMP_th_), especially from the summer samples due to the presence of leafy material, compared with the other substrates (43–54% of the BMP_th_), since steam refining releases carbohydrates, which are inaccessible in the raw wood.

**TABLE 4:** Results for biomethane potentials (BMP) and for biodegradability (BD) of fine sieving rejects, leaves, and liquor fractions from steam refining using different severities (log R_0_) and the dry matter (DM) content of the substrate (without inoculum) (SD in parenthesis).

		Raw material	Severity	BMP_th_ ^a^	BMP_VS_ ^b^	BMP_FM_ ^c^	BD_th_ ^d^	BD_VS_ ^e^	DM
		—	Log R_0_	ml (CH_4_) g (VS)^−1^	ml (CH_4_) g (VS)^−1^	ml (CH_4_) g (FM)^−1^	% of th. BMP	% of initial VS	(%)
Reject	Summer	Mixture	—	550	296 (10)	151 (5)	54	50	54.4
Leaves	Summer	Mixture	—	511	220 (6)	102 (3)	43	36	49.3
		Linden	—	505	269 (11)	115 (5)	53	44	45.8
		Maple	—	518	216 (25)	106 (12)	52	36	52.4
		Oak	—	511	226 (10)	109 (5)	44	37	51.1
Liquor	Summer	—	2.5	367	263 (5)	4.8 (0)	72	—	2.0
		—	3.4	393	286 (4)	5.6 (0)	73	—	2.1
		—	4.0	413	307 (5)	7.0 (0)	74	—	2.4
	Winter	—	2.5	418	235 (7)	2.4 (0)	56	—	1.1
		—	3.4	474	278 (6)	4.1 (0)	59	—	1.6
		—	4.0	440	311 (0)	5.9 (0)	71	—	2.0

Note. The test termination criterion (see the *Biochemical Methane Potential and Biodegradability* section) was achieved latest after 71 days for all tested substrates; however, all tests were run for this period.

a BMP_th_ is the theoretical BMP calculated based on the substrate’s elementary composition considering the Buswell equation (Boyle, 1977).

b BMP_VS_ is the experimental determined BMP related to the VS content of the substrate.

c BMP_FM_ is the experimental determined BMP related to the fresh matter content of the substrate.

d BD_th_ is the ratio of BMP_VS_ to BMP_th_.

e BD_VS_ is the mass-based ratio of substrate biogas produced to initial substrate VS added.

The BMP_VS_ of the different leaves are very close to each other, with linden on the top, but the one of fine sieving reject (296 ml of CH_4_/g VS) is higher than that of all leaf types. It is assumed that it is mainly influenced by leaves, since the other possible shares are nonbiodegradable (ash), hardly biodegradable (bark, wood), or are very low in amount (seed). It is further assumed that during sieving, an easily biodegradable leaf fraction is enriched in fine reject, since a leaf consists of different parts (e.g., leaf apex, margins, veins). When comparing the BMP_VS_ of the different liquors (235–311 ml of CH_4_/g VS), the summer and winter samples were close to each other. In general, the winter samples show a broader range when compared with the summer samples. With increasing severity, the BMP_VS_ increased for both types, even though the amount of microbial inhibitors (such as furfural and 5-HMF) found in the liquor increased (*Composition of the Material Fractions after Steam Refining*). One possible explanation is that while the amount of microbial inhibitors increases at high treatment severities, the overall amount is still quite low, as previous research shows that much higher concentrations of furans are needed to significantly inhibit the biogas production (Janzon et al., 2014). Another possible explanation is a puffering effect of the used inoculum. The fresh matter-related BMP_FM_ showed a clear trend, which correlates with the DM of the samples. Due to its higher DM (54.4%) and also BMP_VS_, the fine sieving reject showed clearly the highest BMP_FM_ (151 ml of CH_4_/g FM). In contrast, DM in liquors was very low (1.1–2.4%). The liquid mainly originates from steam condensation after steam-refining treatment. It is equipment specific, and the DM contents might be higher in upscaled processes.

Overall, the investigated substrates showed good potentials for biomethane production. Values of BMP_VS_ were in a close range between 216 and 311 ml of CH_4_ gVS^−1^ for all investigated substrates. The main difference lies in the water content (based on fresh matter), which is in a range of about 50% for the leaves and fine rejects and above 97% for the liquors. Since emulsifiers as high-value added products can also be produced from the liquor, this pathway should be preferred. During emulsifier production, an insoluble fraction remains, which contains organic matter (Supplementary Data File). Fine rejects and liquor or liquor fractions should be treated together in an anaerobic wet fermentation process. Precondition of wet fermentation is the pumpability of the substrate [water content ≥85% (FNR, 2021)]. This can be realized by mixing of the residue streams. While the fine rejects from sieving and washing are too dry, the optimal water content of the substrate can be realized by mixing with residue streams from washing and purification for emulsifier production. These resulting wastewaters contain VS, which are anaerobically nondegradable due to the large share of noncarbohydrate fractions (Supplementary Data File). However, they are suitable for utilization as process water, which reduces fresh water consumption as well as required wastewater treatment.

### Composting

Composting is a well-researched and practically applied process for converting biodegradable waste into compost under aerobic conditions. Prerequisites are a suitable water content and a porous material structure that allows aeration. The composting of residual streams from the discussed biorefinery concept has not been investigated experimentally, since the laboratory tests yielded only small material amounts. However, appropriate background knowledge on composting is available to develop a theoretical scenario. The evaluation for the summer pruning material is included in the Supplementary Material.

The preparation of the pruning material by screening produced fine and coarse rejects. Furthermore, also washing produced fine rejects, however, in rather low amounts. All of these streams are suitable for composting due to their high amounts of VS. Since coarse rejects are a good structural material for composting, fine rejects are better suitable for anaerobic digestion due to their high BMP (see *Biomethane Potential and Biodegradability*). After anaerobic digestion, a digestate remains, which still is high in VS (84% in DM) and water content (93.4%). If mixed with the coarse rejects (water content 43.9%), the composting mixture reaches a water content of 82.1%. Since the optimum composting water content ranges between 45 and 70% (Körner, 2008), the water content must be adjusted. In the scenario, it was adjusted to 70%. In practice, it can be done by multiple ways, e.g., by applying another sieving protocol resulting in a higher amount of coarse rejects or the addition of further co-substrates such as garden wastes. Also, air drying of coarse rejects can be an option or using excess heat from steam refining for drying.

Besides substrate composition, the organic matter loss during composting depends on the composting conditions (duration, aeration, turning). The potential organic matter loss was calculated based on the chemical composition of the rejects and the insoluble fraction. Based on Deipser (2014), 70% of degradability for hemicelluloses, 50% for cellulose, and 0% for the lignin- and ash-rich residue were assumed. As a result, 26% of the original DM of the pruning material can be found in the compost product.

### Material Flow Analysis

**FIGURE 5:**
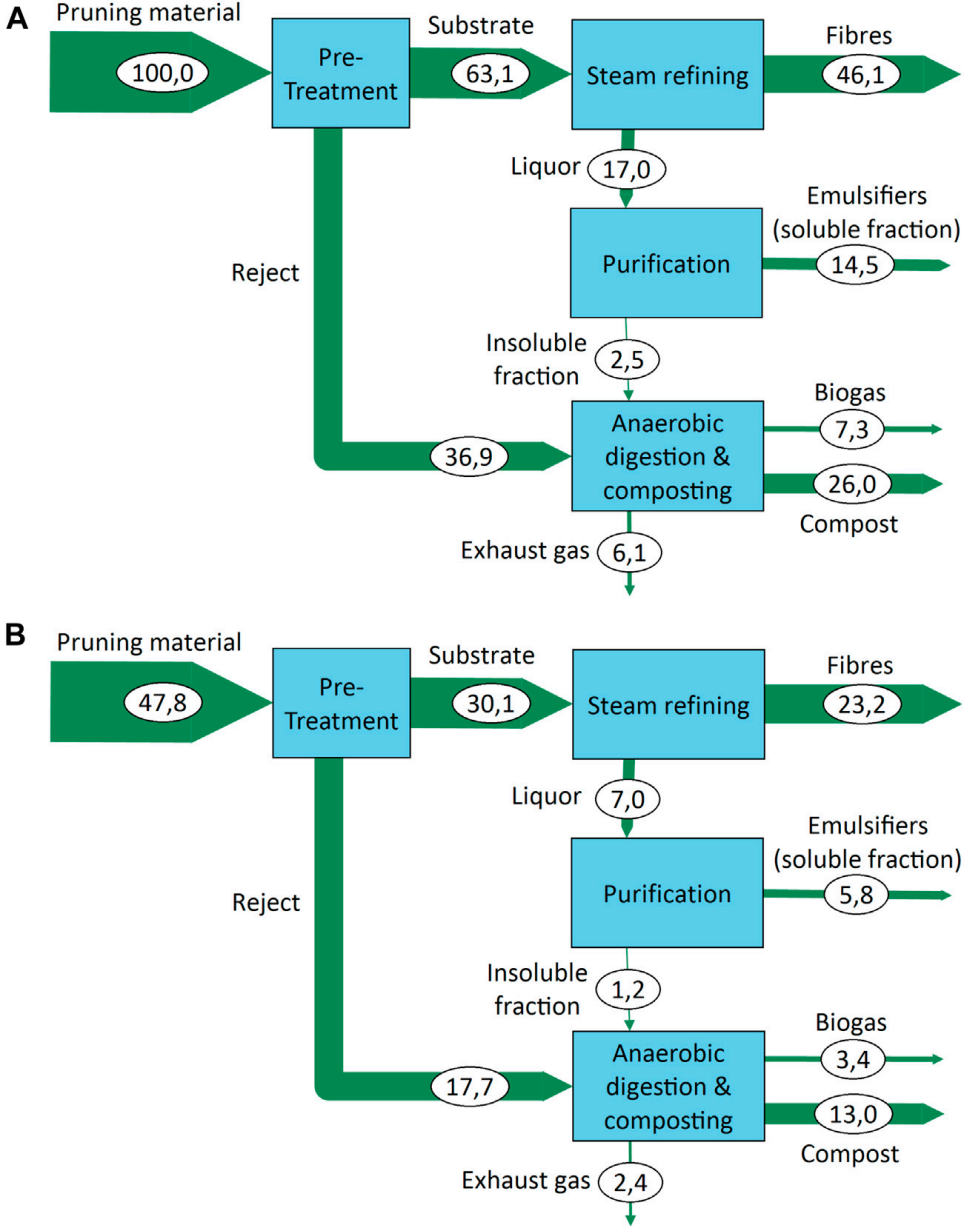
Sankey diagram of the material flow analysis of the studied process network with traced dry matter **(A)** and carbon **(B)**.

The material flow analysis (MFA) shows the pathway of material components within the suggested biorefinery (calculations can be found in the Supplementary Data File). Figure 5 illustrates the pathway of total DM and carbon (C) for the leaf-rich summer pruning material. With the choices made for the preparation, 63.1% of the DM is accepted as substrate for the subsequent steam refining process. The remaining 36.9% of the material is suitable for utilization through anaerobic digestion (see the *Biomethane Potential and Biodegradability* section) in combination with composting (see *Composting* section). In comparison, the numbers for accept and reject of the winter material were 68.8 and 31.2%, respectively.

In the accepted material for steam refining, the leaf matter content of the summer material was only slightly reduced by the pretreatment from 16.2 to 14.8%. However, as shown in the *Paper Test Sheet Properties* section, no significant negative effect on the fiber quality from leaf matter in the substrate could be observed. The steam-refining settings in the shown MFA was chosen for the highest fiber quality produced with a severity of 3.7. Under these conditions, the fiber yield was determined with 46.1% of the original DM content of the summer pruning material. For a conservative estimation of the purification efficiency, values from the purification by filtration were chosen in this scenario with 85.5% yield of the input DM as emulsifiers. The segregated 14.5% are utilized together with the rejects from pretreatment for anaerobic digestion combined with composting.

As discussed in the *Biomethane Potential and Biodegradability* section, the fine rejects from sieving and washing as well as the insoluble fraction from purification were considered for anaerobic digestion. For composting, the resulting digestate was mixed with the coarse reject from sieving as structure material as described in the *Composting* section. This combination results in 7.3 kg of biogas and 26.0 kg of compost for every 100 kg of pruning material DM at the input.

The carbon MFA (Figure 5B) illustrates how carbon is fixated in products. For every 100 kg of DM of pruning material, 47.8 kg of carbon is processed of which 42.2 kg of carbon can be considered as sequestrated in material products. From biogas, 3.3 kg is utilized as methane, and 2.3 kg is emitted as CO_2_.

## Conclusion

To advance the circular economy approach, a holistic utilization of urban tree pruning material within a zero-waste biorefinery is presented in this paper. The analyzed pruning residues differ considerably from conventional stem wood as it contains a large share of twigs, branches, and bark. Due to the anatomical differences, a high amount of extractives and a low amount of carbohydrates were determined for the tree pruning residues. Using steam refining at severities of 2.5–4.0, it was possible to fractionate the tree pruning residues into lignin-containing fibers and an oligosaccharide-rich liquid fraction. At the highest severity, a fiber yield of around 66% and a liquor yield of 26–30% were determined. Different processing pathways for these fractions were evaluated in this work as finding a suitable use will be a key factor in a successful valorization of the pruning residues.

For the fibers, an application as filler material in recycled packaging paper applications might be a viable valorization pathway going forward, as the brightness, tear, and tensile strength of the produced test sheets is low, but the compression strength is comparable with recycled pulp. Future investigations may include mixing trials between recycled pulp and steam-refined tree pruning residue fibers to investigate possible synergistic effects. From the liquor, it was possible to produce emulsions that showed good stability for 8 weeks; however, further research is needed (e.g., long-term stability) using different concentrations and purification steps. The liquors and rejected materials were successfully used for biogas production, and the biomethane contained in the biogas could be used to cover part of the energy demand of the biorefinery, while a utilization of the rejected and nondegradable material for compositing seems feasible. Still, biostability testing of the compost is needed to validate the theoretical scenario presented. In the evaluated range of treatment severities, a high severity led to superior paper properties and a higher liquor content at the cost of a reduction of fiber yield. The differences between summer and winter samples after cleaning and fractionation were found to be small for the paper strength and emulsifying properties. Thus, a year-round utilization in a zero-waste biorefinery seems possible and could enable an improved valorization of tree pruning residues. For a successful commercialization, next to the performance of the materials, the price is of utmost importance. The production cost will depend greatly on upscaling potential, while the achievable market price will depend on final practical applications of the fractions. The described biorefinery is flexible in the potential process pathways by either combining the production of fibers, oligosaccharides, and biogas or by using all of the nonfiber material directly for biogas production, enabling a certain degree of adaption to market conditions.

## Data Availability

The original contributions presented in the study are included in the article/Supplementary Material. Further inquiries can be directed to the corresponding author.

## Supplementary Material

Supplementary Figure 1 Morphology of the fibrous material after steam refining (summer sample, 741 air dried)
