# Supplementary figures and images for: Valorization of Urban Street Tree Pruning Residues in Biorefineries by Steam Refining: Conversion Into Fibers, Emulsifiers, and Biogas
Source: Front Chem. 2021 Nov 15;9:779609. doi: 10.3389/fchem.2021.779609 (PMC8634610; doi:10.3389/fchem.2021.779609)

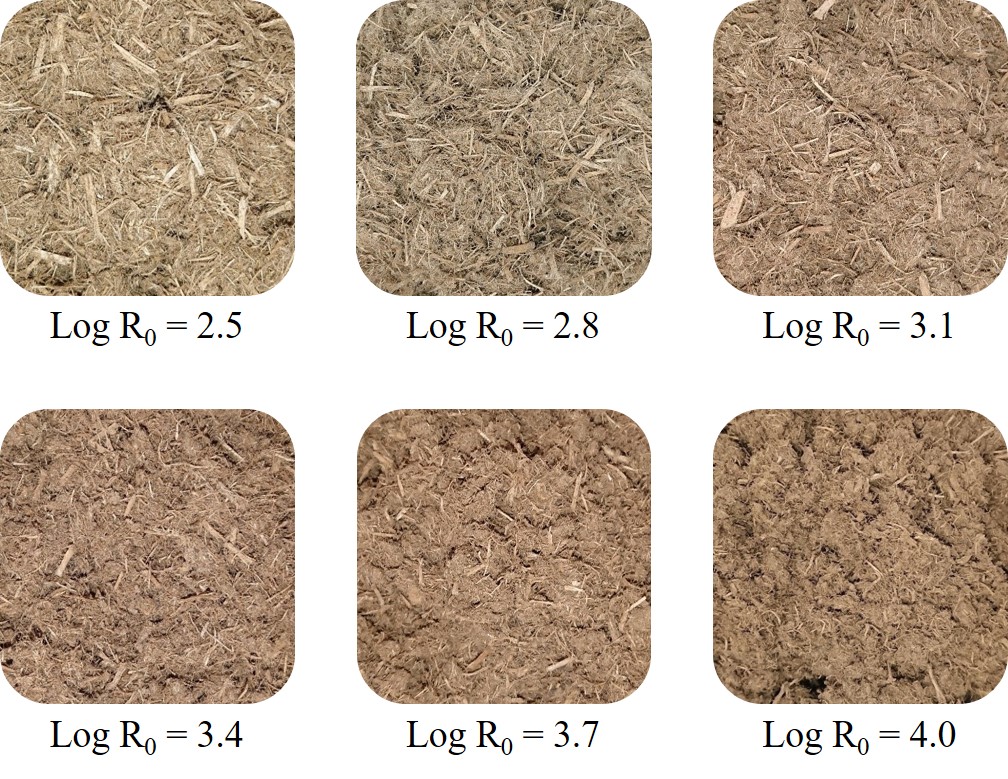

Supplement: Supplementary file 1 [file Image1.JPEG]
